# Supplementary material for: Impact damage reduction of woven composites subject to pulse current
Source: Nat Commun. 2023 Aug 19;14:5046. doi: 10.1038/s41467-023-40752-6 (PMC10439925; doi:10.1038/s41467-023-40752-6)
Supplement: Supplementary file 1 — Supplementary Information [file 41467_2023_40752_MOESM1_ESM.pdf]

**Supplementary Information**

# Impact damage reduction of woven composites subject to pulse current

Yan Li[1], Fusheng Wang[1*], Chenguang Huang[1*], Jianting Ren[1], Donghong Wang[2], Jie Kong[3], Tao Liu[4], Laohu Long[5,6]

1. School of Mechanics, Civil Engineering and Architecture, Northwestern Polytechnical University, 710129 Xi'an, PR China.
2. Shanxi Key Laboratory of Electromagnetic Protection Material and Technology, The 33th Institute of China Electronics Technology Group Corporation, 030032 Taiyuan, PR China.
3. Shaanxi Key Laboratory of Macromolecular Science and Technology, School of Chemistry and Chemical Engineering, Northwestern Polytechnical University, 710072 Xi'an, PR China.
4. School of Engineering and Materials Science, Queen Mary University of London, Mile End Road, London, E1 4NS, UK.
5. State Key Laboratory of Long-Life High Temperature Materials, 618000 Deyang, PR China.
6. Dongfang Electric Corporation Dongfang Turbine Co.,LTD, 618000 Deyang, PR China.

Email: fswang@nwpu.edu.cn; huangcg@nwpu.edu.cn

## Supplementary Figures

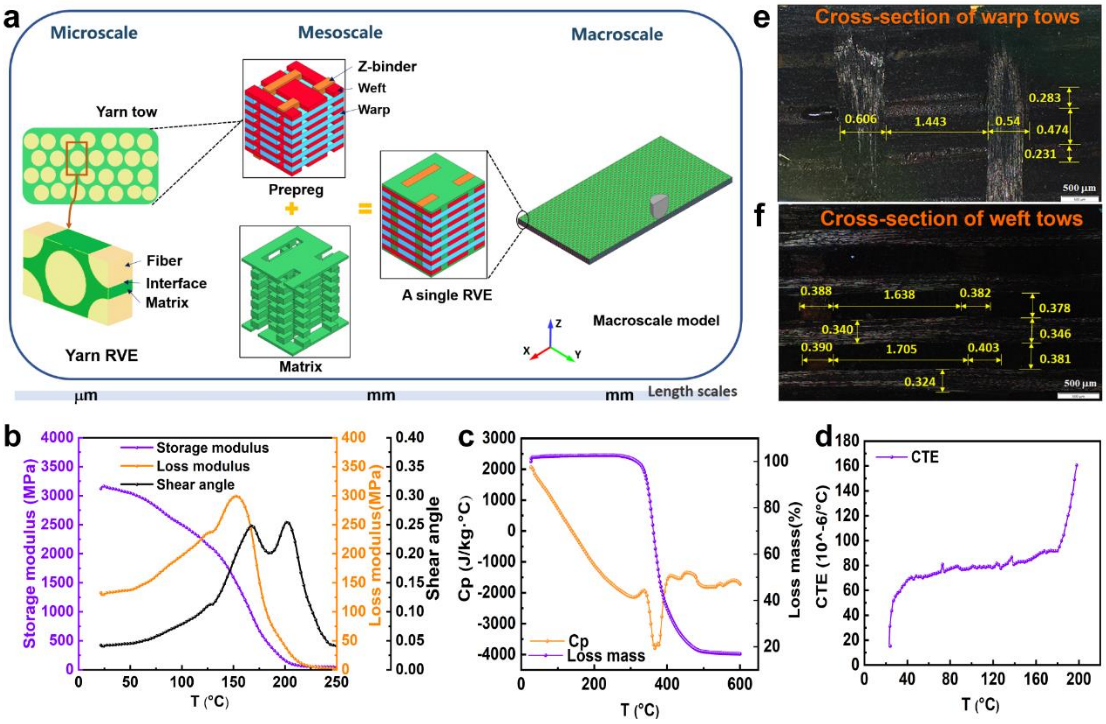


**Supplementary Fig. 1** Multi-scale modeling for 3D orthogonal woven composites. **a** Hierarchical decomposition of multi-scale woven structures. **b-d** Storage modulus, loss modulus, shear angle, specific heat capacity, loss mass, and thermal expansion coefficient as a function of temperature for epoxy resin. **e**, **f** Optical image of cross-sections and geometric parameters. Source data are provided as a Source Data file.

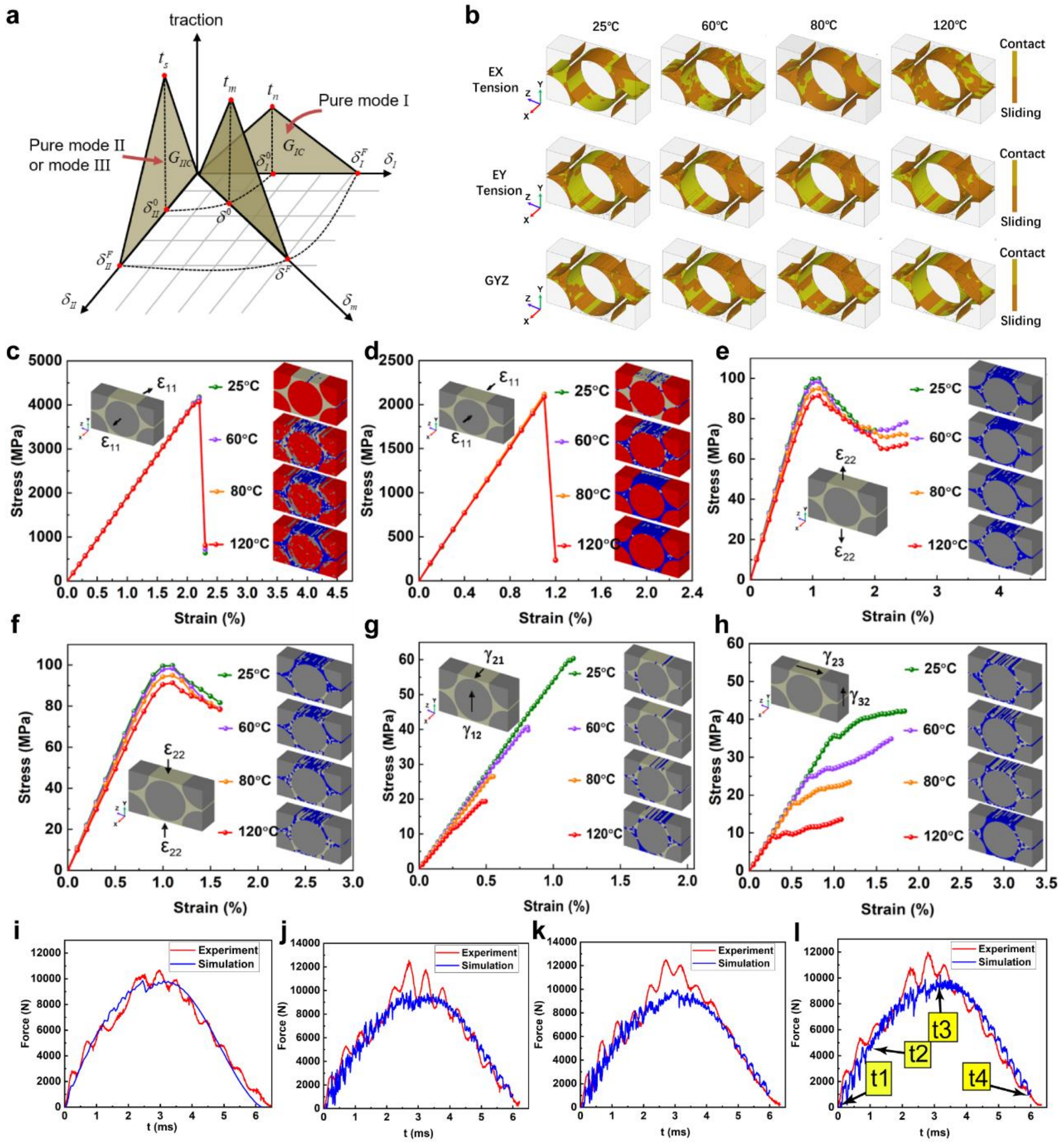


**Supplementary Fig. 2.** Response of microscale RVE at 25℃, 60 ℃, 80℃ and 120℃ and multi-scale model validation for 3D orthogonal woven composites. **a** Mixed-mode bilinear cohesive zone model for fiber-matrix interface. $\delta_i^0$ and $\delta_i^F$ ( $i = \mathrm{I, II, III}$ ) are separation displacements at the maximum cohesive traction and completion of debonding, respectively. $G_{ic}$ ( $i = \mathrm{I, II, III}$ ) denotes fracture toughness. $t_n$ , $t_s$ and $t_m$ are interfacial normal strength, shear strength and mixture strength, respectively. **b** Interface damage evolution of fiber/matrix for microscale RVE. Stress-strain curves and failure modes of microscale RVE. **c** longitudinal tension, **d** longitudinal compression, **e** transverse tension, **f** transverse compression, **g** in-plane shear, **h** out-plane shear. $\varepsilon_{ij}$ and $\gamma_{ij}$ $(i,\ j = 1,\ 2,\ 3)$ are normal strain and shear strain, respectively. **i-l** Comparison of numerical and experimental force curves under impact energy of 50 J and different pulse current peaks 0 A, 30 A, 70 A and 110 A. Source data are provided as a Source Data file.

# Supplementary Tables

**Supplementary Table 1** Properties of the carbon fiber.

| T700-12K Carbon Fibers | Value |
|---|---|
| Longitudinal elastic modulus $E_{f,1}$ (GPa) | 230 |
| Transverse elastic modulus $E_{f,2}$ (GPa) | 14 |
| Poisson's ratio $\upsilon_{f,12}$ | 0.25 |
| Poisson's ratio $\upsilon_{f,23}$ | 0.3 |
| Shear modulus $G_{f,12}$/$G_{f,13}$ (GPa) | 9 |
| Shear modulus $G_{f,23}$ (GPa) | 5 |
| Ultimate tensile strength $X_{f,t}$ (MPa) | 4900 |
| Ultimate compressive strength $X_{f,c}$ (MPa) | 2470 |
| Density $\rho_f$ (kg/m$^3$) | 2400 |
| Longitudinal coefficient of thermal expansion $\alpha_{f1}$ ($10^{-6}$/℃) | -0.5 |
| Transverse coefficient of thermal expansion $\alpha_{f2}$ ($10^{-6}$/℃) | 10.2 |
| Longitudinal thermal conductivity $\lambda_{f1}$ (W/m.K) | 11 |
| Transverse thermal conductivity $\lambda_{f2}$ (W/m.K) | 1.3 |
| Specific heat capacity $C_p$ (J/kg.K) | 750 |

**Note:** Elastic modulus $E$, poisson's ratio $\upsilon$, shear modulus $G$, ultimate strength $X$, density $\rho$, coefficient of thermal expansion $\alpha$, thermal conductivity $\lambda$, specific heat capacity $C_p$.

**Supplementary Table 2** Kink force $F_d$ and maximum pull-out force $F_{max}$ under different test temperatures.

| Temperature | 25 ℃ | 60 ℃ | 80 ℃ | 120 ℃ |
|---|---|---|---|---|
| Kink force $F_d$ (mN) | 75 | 51 | 46 | 23 |
| Maximum pull-out force $F_{max}$ (mN) | 92 | 69 | 64 | 26 |

**Supplementary Table 3** Inter-phase properties.

| Temperature | 25 ℃ | 60 ℃ | 80 ℃ | 120 ℃ |
|---|---|---|---|---|
| Penalty stiffness *K* (MPa) | 10000 | 10000 | 10000 | 10000 |
| Transverse strength $\sigma_{max}$ (MPa) | 60.50 | 44.79 | 41.71 | 18.12 |
| Shear strength $\tau_{max}$ (MPa) | 60.50 | 44.79 | 41.71 | 18.12 |
| Mode I fracture toughness $G_{IC}$ ( J/m$^2$) | 4.9616 | 2.4433 | 2.1979 | 0.6555 |
| Mode II & III fracture toughness $G_{IIC} = G_{IIIC}$ ( J/m$^2$) | 4.9616 | 2.4433 | 2.1979 | 0.6555 |
| Power law exponent for mixed-mode debonding $\alpha$ | 1.20 | 1.20 | 1.20 | 1.20 |

**Note:** Penalty stiffness *K*, transverse strength $\sigma_{max}$, shear strength $\tau_{max}$, Mode I fracture toughness $G_{IC}$, Mode II & III fracture toughness $G_{IIC} = G_{IIIC}$, Power law exponent for mixed-mode debonding $\alpha$ .

**Supplementary Table 4** Mechanical parameters of yarn.

| | $E_{11}$ (GPa) | $E_{22}$ (GPa) | $\upsilon_{12}$ | $\upsilon_{23}$ | $G_{12}$ (GPa) | $G_{23}$ (GPa) | $\alpha_{y1}$ ($10^{-6}$/℃) | $\alpha_{y2}$ ($10^{-6}$/℃) |
|---|---|---|---|---|---|---|---|---|
| Rule of mixture | 190.137 | 10.597 | 0.268 | 0.309 | 5.547 | 3.820 | - | - |
| FEM | 190.634 | 10.829 | 0.275 | 0.312 | 5.694 | 3.671 | 0.389 | 21.84 |
| | $X_T$ (GPa) | $X_C$ (GPa) | $Y_T$ (GPa) | $Y_C$ (GPa) | $S_{12}$ (GPa) | | | |
| Chamis model | 4.039 | 2.036 | 0.075 | 0.075 | 0.056 | | | |
| FEM | 4.169 | 2.118 | 0.074 | 0.081 | 0.060 | | | |

**Note:** Elastic modulus *E*, poisson's ratio $\upsilon$, shear modulus *G*, coefficient of thermal expansion $\alpha$, longitudinal ultimate strength *X*, transverse ultimate strength *Y*, shear strength *S*.

**Supplementary Table 5** Electrical conductivity parameters of carbon fiber.

| Fiber diameter $d_f$ (μm) | Fiber conductivity $\sigma_f$ (S/m) | Fiber modulus $E$ (GPa) | Processing pressure $P$ (MPa) |
|---|---|---|---|
| 7 | $6.25\times10^4$ | 230 | 0.8 |

**Note:** Fiber diameter $d_f$ , Fiber conductivity $\sigma_f$, Fiber modulus $E$, Processing pressure $P$.

**Supplementary Table 6** Electrical conductivity parameters of yarn with carbon fiber volume fraction 82.43%.

| Electrical conductivity | | Equivalent permeability | |
|---|---|---|---|
| $\sigma_{11}$(S/m) | $\sigma_{22}$, $\sigma_{33}$(S/m) | $\mu_{11}$ | $\mu_{22}$, $\mu_{33}$ |
| $5.27\times10^4$ | 1167.8 | $1\mu_0$ | $1\mu_0$ |

**Note:** Electrical conductivity $\sigma$, equivalent permeability $\mu$.

**Supplementary Table 7** Experimental and finite element analysis results of the maximum impact forces.

| Mode | Experimental results (N) | Finite element analysis results (N) | Error (%) |
|---|---|---|---|
| 50 J-0 A | 10750 | 9932 | 7.61 |
| 50 J-30 A | 12313 | 10074 | 18.18 |
| 50 J-70 A | 12067 | 10038 | 16.81 |
| 50 J-110 A | 12476 | 10259 | 17.77 |
